# Acetylcholine Acts through Nicotinic Receptors to Enhance the Firing Rate of a Subset of Hypocretin Neurons in the Mouse Hypothalamus through Distinct Presynaptic and Postsynaptic Mechanisms^1^,^2^

**DOI:** 10.1523/ENEURO.0052-14.2015

**Published:** 2015-03-06

**Authors:** Wen-Liang Zhou, Xiao-Bing Gao, Marina R. Picciotto

**Affiliations:** Departments of Psychiatry and Comparative Medicine, Kavli Insitute for Neuroscience, Yale University School of Medicine, New Haven, Connecticut 06508

**Keywords:** acetylcholine, hypocretin, nicotine, presynaptic

## Abstract

Hypocretin/orexin neurons regulate many behavioral functions, including addiction. Nicotine acts through nicotinic acetylcholine receptors (nAChRs) to alter firing rate of neurons throughout the brain, leading to addiction-related behaviors. While nAChRs are expressed in the hypothalamus and cholinergic fibers project to this structure, it is unclear how acetylcholine modulates the activity of hypocretin neurons. In this study, we stimulated hypocretin neurons in mouse brain slices with ACh in the presence of atropine to dissect presynaptic and postsynaptic modulation of these neurons through nAChRs. Approximately one-third of tested hypocretin neurons responded to pressure application of ACh (1 mM) with an increase in firing frequency. Stimulation of postsynaptic nAChRs with ACh or nicotine resulted in a highly variable inward current in approximately one-third of hypocretin neurons. In contrast, ACh or nicotine (1 µM) reliably decreased the frequency of miniature EPSCs (mEPSCs). Antagonism of nAChRs with mecamylamine also suppressed mEPSC frequency, suggesting that an endogenous, tonic activation of presynaptic nAChRs might be required for maintaining functional mEPSC frequency. Antagonism of heteromeric (α4β2) or homomeric (α7) nAChRs alone suppressed mEPSCs to a lesser extent. Finally, blocking internal calcium release reduced the frequency of mEPSCs, occluding the suppressive effect of presynaptic ACh. Taken together, these data provide a mechanism by which phasic ACh release enhances the firing of a subset of hypocretin neurons through postsynaptic nAChRs, but disrupts tonic, presynaptic nAChR-mediated glutamatergic inputs to the overall population of hypocretin neurons, potentially enhancing the signal-to-noise ratio during the response of the nAChR-positive subset of neurons.

## Abstract (precis)

Neurons expressing the neuropeptide hypocretin regulate many behavioral functions, including sleep, motivation, and behaviors related to addiction. The ability of nicotine to stimulate nicotinic acetylcholine receptors (nAChRs) is essential for its addictive properties, but little is known about whether, and how, nicotine and the endogenous neurotransmitter acetylcholine affect hypocretin neurons.

## Significance Statement

Neurons expressing the neuropeptide hypocretin regulate many behavioral functions, including sleep, motivation, and behaviors related to addiction. The ability of nicotine to stimulate nicotinic acetylcholine receptors (nAChRs) is essential for its addictive properties, but little is known about whether, and how, nicotine and the endogenous neurotransmitter acetylcholine affect hypocretin neurons. This study suggests that phasic acetylcholine release can enhance the firing of a subset of hypocretin neurons through postsynaptic nAChRs, while disrupting tonic activation of presynaptic nAChRs necessary for maintaining functional glutamatergic inputs to hypocretin neurons. We propose that this mechanism could enhance the signal-to-noise ratio of the electrical response to nicotine or acetylcholine in the nAChR-positive subset of neurons.

## Introduction

A small group of neurons that express hypocretin (Hcrt, also known as orexin) resides in the perifornical and lateral hypothalamus (de Lecea et al., 1998; Sakurai et al., 1998), and these neurons project throughout the brain and spinal cord (Peyron et al., 1998; van den Pol, 1999; Bayer et al., 2002). Hcrt+ neurons play important roles in modulating multiple behaviors, including circadian rhythmicity (Mileykovskiy et al., 2005), appetite and food intake (Sakurai et al., 1998; Wu et al., 2002), arousal (Boutrel et al., 2010), goal-oriented behaviors (Boutrel et al., 2005), and emotions (Blouin et al., 2013). In addition, a number of studies implicate hypocretin signaling in the rewarding and addictive properties of drugs of abuse (Mahler et al., 2012; Rao et al., 2013; Muschamp et al., 2014), including nicotine (Hollander et al., 2008).

Nicotine addiction is mediated by nicotinic acetylcholine receptors (nAChRs). Histochemical studies have shown that nAChRs are also expressed in hypothalamus (Avissar et al., 1981; Wada et al., 1989; O'Hara et al., 1998). Specifically, Hcrt+ neurons receive appreciable cholinergic innervation arising from basal forebrain (Sakurai et al., 2005; Henny and Jones, 2006), which indicates that the activity of Hcrt+ neurons might be modulated by nAChRs. nAChRs are pentameric, non-selective cation channels. Activation of nAChRs results in a physiological net flow of inward current, which directly depolarizes the cell and generally affects neuronal input and/or output, influencing subsequent behaviors. nAChRs may be located both presynaptically and postsynaptically. Stimulation of these receptors is known to increase neurotransmitter release, and can also depolarize the postsynaptic neuron (Gioanni et al., 1999; Mansvelder et al., 2002; Lambe et al., 2003). Previous studies have shown that nicotine can alter the firing of Hcrt+ neurons in rat as measured by c-fos immunoreactivity (Pasumarthi et al., 2006; Pasumarthi and Fadel, 2008), but it is not known whether nAChRs are expressed on these neurons, whether the effects on c-fos immunoreactivity were due to postsynaptic signaling, presynaptic signaling, or network changes due to changes in other neuronal subtypes. Since the ability of nicotine to alter hypocretin signaling may be important for its addiction liability (Hollander et al., 2008), it is important to understand the cellular mechanisms underlying these physiological effects.

To investigate the effect of nAChR stimulation on function of the hypocretin system, we first investigated how acetylcholine (ACh) in the presence of atropine affects spontaneous action potential firing, which is a measure of nicotinic influence on the output of Hcrt+ neurons. Next, we separated presynaptic and postsynaptic modulation by ACh and nicotine using synaptic blockers, and used pressure application (puff) or fast pipetting of drugs to identify electrophysiological changes following the stimulation of nAChRs. A transient puff of ACh or nicotine at high concentration was used to mimic phasic transmission to determine the postsynaptic response (Alexander et al., 2009). nAChRs can increase release of glutamate from presynaptic terminals in several brain areas, including the ventral tegmental area (Mansvelder et al., 2002) and the prefrontal cortex (Gioanni et al., 1999; Lambe et al., 2003). To assess the effects of cholinergic transmission on the basal level of presynaptic glutamatergic transmission, spontaneous miniature EPSCs (mEPSCs) were recorded following a puff of ACh as well as in response to bath-applied nicotine at a concentration that mimics brain levels during smoking. We show that the activity of both Hcrt+ neurons and the presynaptic glutamatergic terminals projecting to these cells is modulated by nAChR signaling in a manner that appears to enhance the output of a subset of Hcrt+ neurons during phasic ACh signaling.

## Materials and Methods

### Animals

Male and female adult Hcrt-GFP mice (Li et al., 2002) (2–5 months old, backcrossed onto the C57BL6/J genetic background for at least 10 generations) were group housed and maintained on a 12–12 h light–dark cycle with food and water available *ad libitum*. Use of animals was in strict accordance with NIH Care and Use of Laboratory Animals Guidelines.

### Brain slice and electrophysiology

Briefly, mouse brains were harvested following acute decapitation. Brains were immediately immersed in ice cold, oxygenated artificial cerebrospinal fluid (ACSF). ACSF contained (in mM): 125 NaCl, 26 NaHCO_3_, 10 glucose, 2.3 KCl, 1.26 KH_2_PO_4_, 2 CaCl_2_ and 1 MgSO_4_, pH 7.4. After being trimmed to a small tissue block containing the hypothalamus, brain chunks were cut on a vibratome into coronal slices (300 μm). Acute slices were incubated in a holding chamber with protective NMDG ACSF (containing (in mM): 110 N-methy-D-glucamine, 110 HCl, 2.5 KCl, 1.2 NaH_2_PO_4_, 25 NaHCO_3_, 25 glucose, 10 MgSO_4_, 0.5 CaCl_2_, pH 7.4) at 36°C for 30 min, then transferred to regular ACSF and maintained at room temperature (according to the method described in http://www.brainslicemethods.com).

All experimental measurements were performed at 32–34°C. Whole-cell recordings were made from visually-identified, GFP-positive neurons in hypothalamus under voltage (holding *V* = −60 mV) or current clamp configuration. Electrical signals were amplified with a Multiclamp 700B and digitized with Digidata 1440A (Molecular Devices). Micropipettes with a tip resistance of 4–7 MΩ were made of borosilicate glass (Warner Instruments) using a Sutter micropipette puller (P-97) and back filled with an intracellular solution containing (in mM): 135 K-gluconate, 2 MgCl_2_, 10 Na_2_-phosphocreatine, 3 Na_2_-ATP, 0.3 Na_2_-GTP, and 10 HEPES (pH 7.3). Only recordings with stable series resistance (<30 MΩ) were analyzed.

### Drug application

All drugs were dissolved in ACSF. For bath perfusion, D-2-amino-5-phosphonovalerate (AP-5), 6-cyano-7-nitroquinoxaline-2, 3-dione disodium (CNQX), picotoxin (PTX), tetrodotoxin citrate (TTX), and atropine (Sigma) were dissolved in ACSF at their final concentration. For focal pressure application (puff), acetylcholine (1 mM) or nicotine (100 μM) was loaded into a glass micropipette (2 − 4 MΩ), and the pipette tip positioned 40 − 50 μm from the cell body. Drug solutions were pressure-ejected via a computer-driven picospritzer (puff duration: 5 s) during the electrical recording. The picospritzer was used to control the pressure, timing, and duration of the puff. For fast bath application, 200 μL of nicotine (5 μM), mecamylamine (50 μM), dihydro-β-erythroidine hydrobromide (50 μM), methyllycaconitine (50 nM), dantrolene (250 μM), and (−)-xestospongin C (25 μM) were pipetted into the recording chamber (1 mL of volume), at 5× final concentration, carefully along the chamber wall slope.

### Data analysis

Electrical recording data of nAChR currents were analyzed using Clampfit 10 (Molecular Devices). Traces were filtered with Gaussian low-pass with 50 Hz cutoff before current amplitude measurements. Other electrical data were analyzed using Axograph X 1.5.5 (Axograph Scientific). To analyze miniature EPSCs, we took 150 − 200 events for baseline and washout from each cell, during a time period ranging from 60 to 120 s. For Ach, we took all events from within 90 s after application of ACh. Traces were filtered with Gaussian low-pass with 500 Hz cutoff before event searching. A template EPSC was defined with amplitude −20 pA, rise 0.5 ms, and decay 3 ms, and only those EPSCs with amplitude ≥−10 pA were counted. Following programmed event detection, all events were examined by eye to be counted as EPSCs. All statistics were done on raw data. Unpaired Student's *t* tests and chi square (χ^2^) test were used for determining statistically difference. Significance was set as *p* < 0.05, and high significance as *p* < 0.01. Values are presented as mean ± SEM.

## Results

### Nicotinic stimulation boosts spontaneous action potential firing in hypocretin neurons

**Figure 1:**
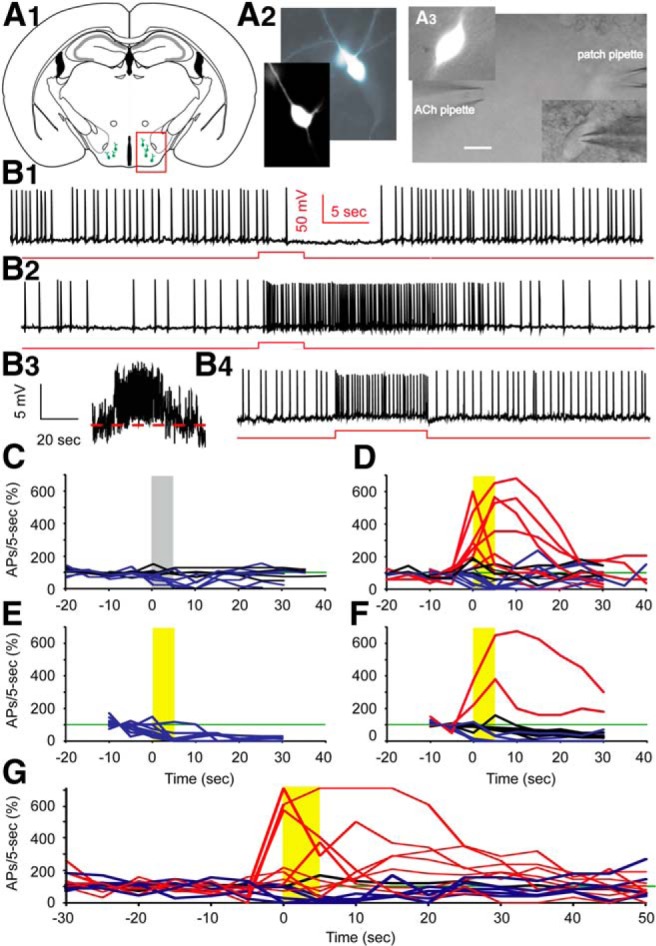
Cholinergic stimulation by ACh boosts spontaneous action potential firing in Hcrt+ neurons. ***A_1_***, Sketch of a brain slice showing Hcrt+ neurons (green cells in the red box) residing in the hypothalamus. ***A_2_***, Morphology of two Hcrt+ neurons shown in the fluorescent channel. ***A_3_***, Differential Interference Contrast video-microscopy showing the experimental paradigm of pressure application (puff) of drug onto the soma and proximal processes, while keeping the patch onto the neuron. Scale bar, 10 µm. ***B_1_***, Mechanical interference (puff of bath solution) frequently produces a temporary depression of spontaneous firing. ***B_2_***, In the presence of atropine (4 µM), application of ACh (1 mM) boosts action potential firing for tens of seconds. ***B_3_***, The same trace as in ***B_2_***, except on a different scale and filtered with Gaussian low pass 5 Hz. ***B_4_***, Injection of +10 pA of current into the Hcrt+ neuron notably increases the firing frequency. Red lines represent the timing and duration of ACh application or current injection. ***C***, Mechanical interference does not affect firing (*n* = 4/11 cells), or results in a temporary depression of firing (*n* = 7/11 cells). ***D***, Differential responses to the puff of ACh. In 7 out of 20 cells, firing was enhanced by ACh, 4 out of 20 cells were not affected, while 9 out of 20 cells were inhibited. ***E***, With DHβE in the bath, ACh did not increase firing of any Hcrt+ neurons tested (*n* = 11/11 cells). ***F***, With MLA in the bath, a puff of ACh boosted firing in 2 of 16 cells, had no effect in 9 of 16 cells, and decreased firing in 5 of 16 cells. The gray bar indicates the time duration of ACSF application; yellow bars indicate the time duration of ACh application. ***C−F***, All experiments were conducted in the presence of atropine. ***G***, The responses of Hcrt+ neurons to ACh in absence of atropine. In 8 out of 14 cells, firing was enhanced by ACh, in 1 out of 14 cells firing was unaffected, while in 5 out of 14 cells firing was inhibited.

To determine whether phasic nicotinic stimulation modulates the electrical output of Hcrt+ neurons, we patched the cells with an ATP-rich (5 mM, vs 3 mM in other experiments) intracellular solution and investigated the effect of pressure-applied ACh on spontaneous action potential firing. An adequate level of intracellular ATP is required for normal Hcrt+ neuron function (Liu et al., 2011), potentially because Hcrt+ neurons are involved in the regulation of energy status (Girault et al., 2012; Gao and Horvath, 2014). In the presence of atropine, Hcrt+ neurons regularly fired action potentials. Mechanical interference as a result of puffing bath solution induced a temporary reduction in firing frequency (<50% of baseline) in 7 out of 11 cells (Fig. 1*B1*,*C*), despite inducing no, or minimal, outward current (<10 pA, data not shown). Firing in 4 out of 11 cells was not altered by puffing bath solution (50% < change < 200% at 0 − 10 s). In contrast, in 7 out of 20 cells, a puff of ACh (1 mM) increased the firing rate (> 200% of baseline) with a significant depolarization of the membrane potential (Fig. 1*B2*,*D*). We note that this experimental design is not suitable to detect decreases in firing due to presynaptic mechanisms. Only blocking all mEPSCs using CNQX+AP-5 induces even a small change in firing rate of a hypocretin neuron (∼2 Hz to ∼1.6 Hz; Li et al, 2002), which is not distinguishable using the current criteria (50% < change < 200%). In nine cells, an ACh puff decreased the firing rate, and in four cells the ACh puff had no effect. Thus, stimulation of nAChRs depolarizes approximately one-third of Hcrt+ neurons and boosts the action potential firing of these cells.

**Figure 2:**
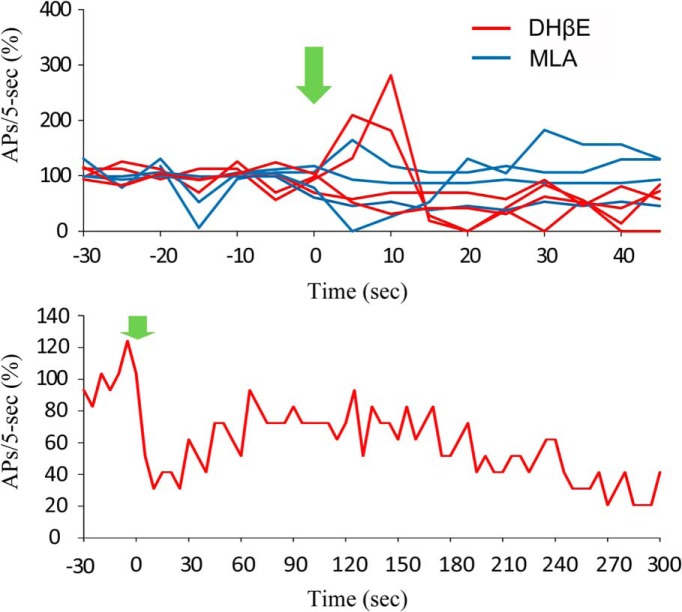
The effects of α4β2 receptor antagonist, DHβE, and α7* receptor antagonist, MLA, on the action potential firing of Hcrt+ neurons. Top, Change of firing rate (action potentials/5 s) before and after bath application of DHβE (red) and MLA (blue). Bottom, A representative cell reduced in firing rate upon bath application of DHβE, and partially recovered over time.

To identify the receptor type(s) that mediate this depolarization and increase in firing rate, we applied an ACh puff in the presence of the heteromeric (α4β2) nAChR antagonist, dihydro-β-erythroidine (DHβE), or the homomeric (α 7) nAChR antagonist, methyllycaconitine (MLA). When applied alone, DHβE significantly decreased the firing rate by 62.9% (*p* = 0.00027) at 10 µM, whereas MLA did not change the firing rate of Hcrt+ neurons at a concentration of 10 nM (Fig. 2, top; 20 − 30 s after puff vs 10 − 20 s before puff: *p* = 0.58). However, after addition of either reagent, most cells recovered to a new stable firing state that lasted for several minutes with attachment of the recording pipette (Fig. 2, bottom). DHβE (10 µM) significantly reduced the ability of ACh to increase firing in 11 of 11 Hcrt+ neurons (χ^2^ test, *p* = 0.0069) and increased the likelihood that an ACh puff could decrease the firing rate (*p* = 6.8E-6, 10 − 20 s after puff; Fig. 1*E*). In contrast, although MLA (10 nM) antagonized the effects of an ACh puff in the majority of Hcrt+ neurons, ACh still increased the firing rate in 2 of 16 cells in the presence of the homomeric nAChR antagonist (χ^2^ test, *p* = 0.00039; Fig. 1*F*). In this data set, some cells increased their firing rate during the ACh puff and returned to normal right after puff cessation, whereas 10 of 17 cells responded with a prolonged period of firing increase. These prolonged periods of firing may be due to increased activity of Hcrt neurons triggering action potentials in a recurrent circuit.

Muscarinic acetylcholine receptors (mAChRs) are expressed by ∼30% of hypocretin neurons (Sakurai et al., 2005), and may contribute significantly to cholinergic modulation of these cells. To determine the overall response of hypocretin neurons to cholinergic inputs, we tested the effect of ACh in the absence of atropine to engage both nAChRs and mAChRs. ACh induced depolarization and increased firing rate in 57% (8 out of 14) of hypocretin neurons (Fig. 1*G*), a significant increase compared to the presence of atropine (χ^2^ test, *p* = 0.0040). The remaining cells showed no effect (1 out of 14 cells) or responded to ACh with decreased firing (5 out of 14 cells).

### Postsynaptic nAChR currents can be induced in one third of hypocretin neurons

**Figure 3:**
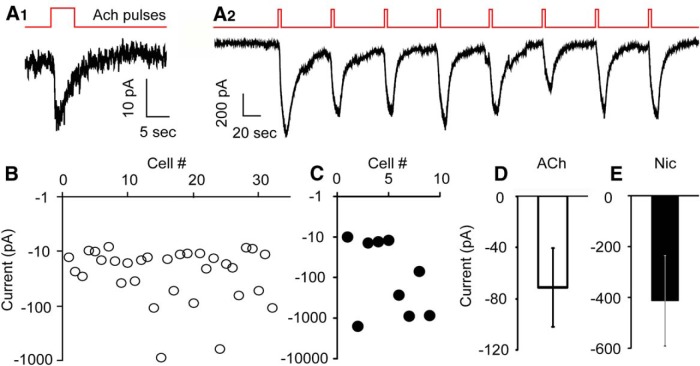
Nicotinic stimulation induced variable inward currents in a third of Hcrt+ neurons. ***A_1_***, A small, desensitizing current evoked by ACh (1 mM) puff, in the presence of atropine (4 µM). ***A_2_***, Large, repetitive currents evoked by ACh (1 mM) puff every 60 s. ***B***, In 32 out of 92 Hcrt+ neurons, an ACh (1 mM) puff evoked inward currents of variable size. ***C***, In 9 out of 29 Hcrt+ neurons, a nicotine (Nic; 100 µM) puff evoked inward currents of variable size. ***D***, ***E***, Mean sizes of currents evoked by puffing ACh (1 mM; ***B***) or nicotine (100 µM; ***C***), respectively, onto Hcrt+ neurons.

Although mRNAs encoding nAChR subunits are expressed in lateral hypothalamus (Clarke et al., 1985; Wada et al., 1989) where Hcrt+ neurons reside, it is not yet clear on which cell types and in what subcellular structure nAChRs are functional, and whether Hcrt+ neurons express nAChRs. To dissect the mechanisms underlying nicotinic stimulation of Hcrt+ neurons, we evaluated postsynaptic nAChRs on Hcrt+ neurons selectively by using synaptic blockers to rule out contributions from glutamate- and GABA-activated currents. We recorded from Hcrt+ neurons in voltage clamp mode in the presence of TTX (0.5 μM), PTX (100 μM), CNQX (10 μM), AP-5 (30 μM), and atropine (4 μM), while puffing 1 mM ACh onto the cell body of the recorded cell. A brief puff of ACh evoked an inward current in about one-third of Hcrt+ neurons (32 out of 92 cells). The peak amplitudes of the inward currents ranged from −10 to −850 pA, with an average size of −71.1 ± 30.9 pA (*n* = 32). We also tested the nAChR agonist nicotine (100 μM). A nicotine puff produced a response similar to that induced by ACh in Hcrt+ neurons. Inward currents were recorded in 9 out of 29 cells, ranging from −10 to −1500 pA, with an average size of −413 ± 178 pA (*n* = 9; Fig. 3). We also wished to determine whether the small currents might be sufficient to alter the function of the Hcrt+ neurons. Injection of a small inward current (−10 pA) markedly increased the spontaneous action potential firing rate to 324% ± 101% (Student’s *t* test, *p* = 1.0E-5, *n* = 3) of the baseline level (Fig. 1*B4*), suggesting that nicotinic currents are likely to have a significant effect on the output of these cells. nAChRs are known to be easily desensitized by agonists (Giniatullin et al., 2005). Consistent with these observations, we observed that postsynaptic nAChR-mediated currents on Hcrt+ neurons were desensitized following 5 s exposure to 1 mM ACh or 100 μM nicotine. Previous studies have shown that most nAChR subtypes, including α4β2, α3β4 and α7, recover from the desensitization induced by brief (1 − 5 s) exposure to 1 mM ACh in 60 s or less (Reitstetter et al., 1999; Meyer et al., 2001; Paradiso and Steinbach, 2003; McCormack et al., 2010). While the majority of currents did not recover rapidly following ACh stimulation, we observed a rare subset of Hcrt+ neurons (3 out of 121 cells) that responded to repeated ACh or nicotine puffs every 60 s (Fig. 3*A2*), which happened only when the current size was greater than 500 pA.

### Acetylcholine and nicotine decrease the frequency of mEPSCs in hypocretin neurons

**Figure 4:**
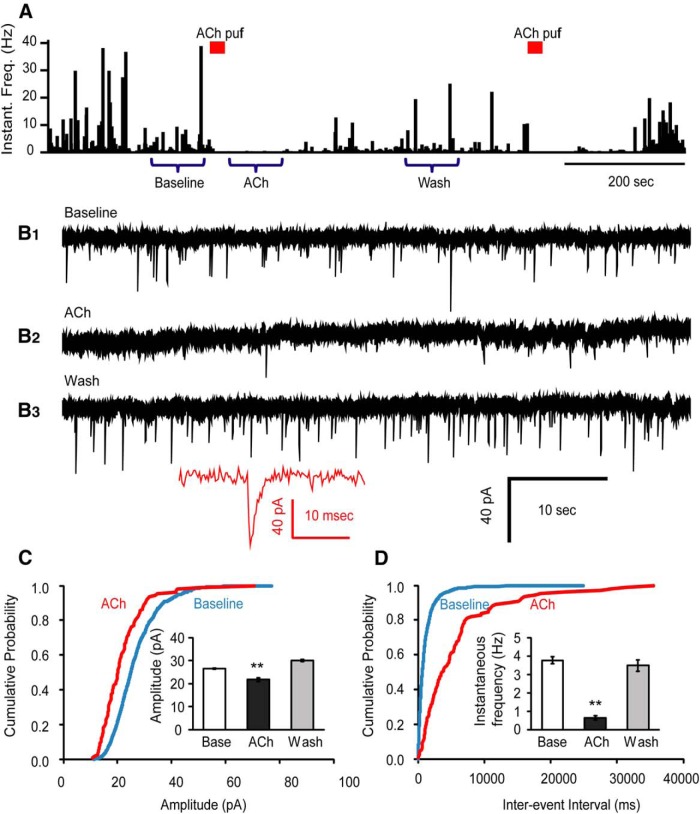
ACh suppresses glutamatergic spontaneous mEPSCs onto Hcrt+ neurons in the presence of atropine. ***A***, Histograms of mEPSC instantaneous frequency show the time course of the effect of ACh on glutamatergic mEPSCs from synapses impinging onto Hcrt+ neurons. Bars above the graph indicate the time point of the ACh (1 mM) puff. The original recordings indicated by “Baseline”, “Ach”, and “Wash” are displayed in ***B_1_***, ***B_2_***, and ***B_3_***, respectively. Inset shows the full trace of an example mEPSC event. ***C***, Cumulative probability plot of the mEPSC amplitude at baseline and upon ACh application from six cells. Inset shows the mean value of the mEPSC amplitude at three time periods: baseline, under the influence of ACh, and washout. **p < 0.01. ***D***, Interevent intervals of mEPSCs at baseline and upon ACh application from six cells (same cells as in ***C***). Inset shows the mean of instantaneous frequency of the mEPSC at three periods of time: baseline, under the influence of ACh, and after washout.

Next, we tested whether nAChRs are expressed in presynaptic glutamatergic terminals that impinge onto Hcrt+ neurons, and what physiological changes might occur to synaptic transmission following presynaptic receptor stimulation. To do this, we applied TTX, PTX, and atropine in the bath and recorded spontaneous mEPSCs from the Hcrt+ neurons. A brief puff of ACh above the tissue surface next to the Hcrt+ neuron reliably decreased mEPSC occurrence for ∼1 min, followed by full recovery (Fig 4*A*,*B*). The average instantaneous frequencies of mEPSCs at baseline, upon ACh application, and after washout were 3.76 ± 0.19 Hz, 0.64 ± 0.12 Hz, and 3.48 ± 0.31 Hz, respectively. The average peak amplitudes of mEPSCs at baseline, upon ACh application, and washout were 26.4 ± 0.3 pA, 21.8 ± 0.8 pA, and 30.0 ± 0.5 pA, respectively. Analysis shows that phasic stimulation of preterminal nAChRs significantly reduced vesicle release probability by 83.0% (*p* = 2.06E-39). The average peak size of mEPSCs was also slightly reduced by 17.4% (*p* = 1.88E-7; Fig. 4*C*,*D*).

**Figure 5:**
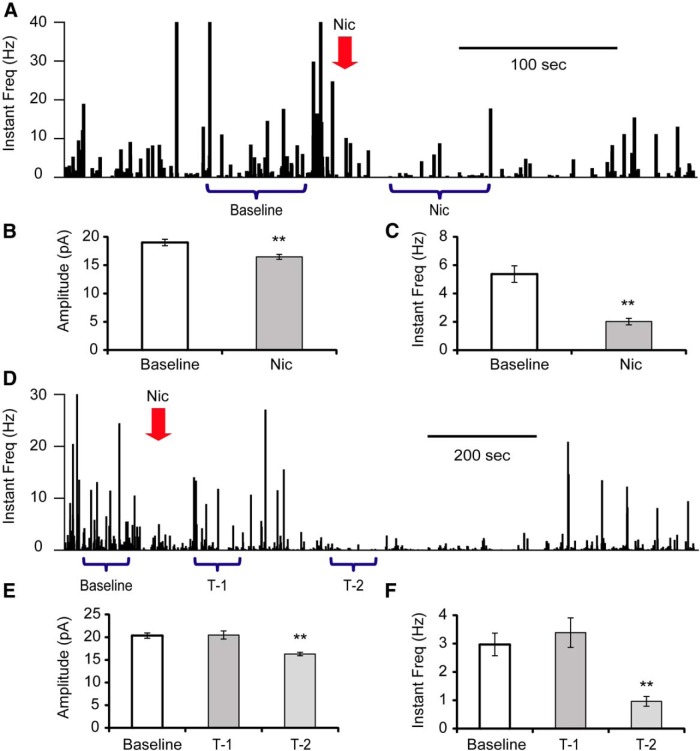
Effects of nicotine exposure on glutamatergic mEPSCs onto Hcrt+ neurons. ***A***, Histograms of mEPSC instantaneous frequency showing the effect of nicotine (Nic) exposure (100 µM) on mEPSCs onto Hcrt+ neurons. The arrow indicates the time of nicotine application. Depending on the response pattern, two periods of time (baseline and Nic) were used for analysis. ***B***, ***C***, Mean value of amplitude (***B***) and instantaneous frequency (***C***) at baseline and under the influence of nicotine. *n* = 4 of 11 cells. **p < 0.01. ***D***, Histograms show another representative response to nicotine (1 µM) on mEPSCs onto an Hcrt+ neuron. Three periods of time (baseline, T-1, and T-2) were used for analysis. ***E***, ***F***, Mean value of amplitude (***E***) and instantaneous frequency (***F***) of mEPSCs during baseline recording and at two periods after nicotine exposure. *n* = 4 of 11 cells.

Nicotine is a strong exogenous agonist of nAChRs, and also a prevalent drug of addiction. During cigarette smoking, nicotine concentration increases rapidly in the brain (Berridge et al., 2010; Rose et al., 2010). To mimic this fast onset of brain nicotine concentration, we pipetted 5× nicotine solutions into the recording chamber within 5 s, to a final concentration of 1 μM (Henningfield et al., 1993). In 4 out of 11 tested cells, the mEPSC frequency was immediately depressed from 5.37 ± 0.59 Hz to 2.02 ± 0.23 Hz (Fig. 5*A−C*). In an additional four cells, the immediate effect of nicotine was not significant, with frequency changed from 2.97 ± 0.40 Hz to 3.39 ± 0.52 Hz (*p* = 0.527; Fig. 5*D−F*, T-1), but following this initial 1 − 2 min period, suppression of mEPSC frequency (0.96 ± 0.17 Hz, *p* = 6.8E-6) was observed for 1 − 2 min (Fig. 5*D**−*F, T-2). Finally, there were three cells with no obvious response to nicotine exposure.

### Antagonists of nAChRs mimic the effect of agonist application on mEPSC frequency

**Figure 6:**
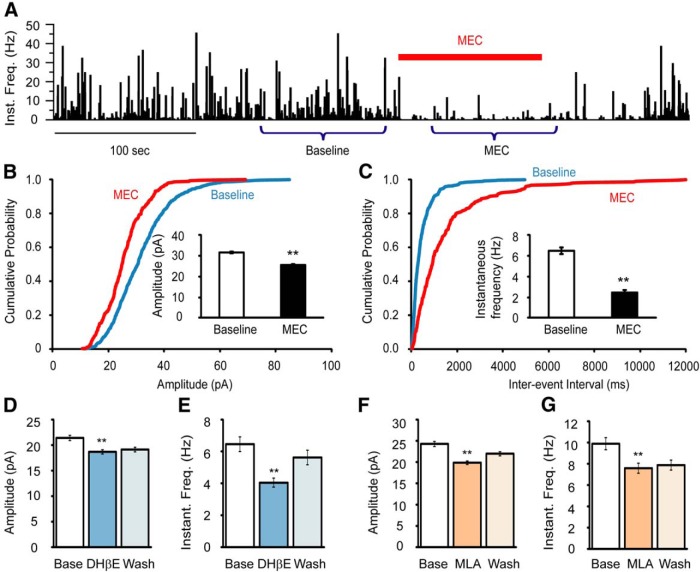
Nicotinic AChR antagonists suppressed glutamatergic mEPSCs onto Hcrt+ neurons. ***A***, Mecamylamine, a nonselective nAChR antagonist, strongly suppressed mEPSCs onto Hcrt+ neurons. The bar above the graph indicates the duration of MEC (10 µM) exposure. Miniature EPSCs from two periods of time (Baseline and MEC) were used for analysis. ***B***, Cumulative probability plot of the mEPSC amplitude at baseline and during MEC exposure was made from the recordings of four cells. Inset shows the mean values of the mEPSC amplitude at baseline and following MEC exposure. **p < 0.01. ***C***, Interevent intervals of mEPSCs at baseline and upon MEC application from four cells (same cells as in ***C***). Inset shows the mean instantaneous frequency of the mEPSCs at baseline and following MEC exposure. ***D***, ***E***, DHβE, an antagonist of heteromeric nAChRs (particularly α4β2 nAChRs), moderately suppressed the amplitude (***D***) and instantaneous frequency (***E***) of mEPSCs onto Hcrt+ neurons. *n* = 7 cells. ***F***, ***G***, MLA, a relatively selective α7 nAChR antagonist, partially suppressed mEPSC amplitude (***F***) and instantaneous frequency (***G***). *n* = 8 cells.

Under physiological conditions, activation of nAChRs induces excitatory inputs by evoking inward currents. The suppressive effect on mEPSCs might therefore be due to desensitization of nAChRs. To test this possibility, we applied mecamylamine (MEC), a nonselective antagonist of nAChRs. The application of MEC alone caused a substantial suppression of mEPSCs (Fig. 6), with instantaneous frequency decreased to 37.6% (from 6.49 ± 0.32 Hz to 2.44 ± 0.24 Hz, *p* = 1.82E-23), and amplitude decreased to 80.7% (from 31.6 ± 0.4 pA to 25.5 ± 0.4 pA, *p* = 1.47E-22) of baseline values. Furthermore, both the heteromeric (predominantly α4β2) nAChR antagonist DHβE (10 µM) and the homomeric, α7 nAChR antagonist MLA (10 nM) partially reduced event frequency to 62.5% (from 6.45 ± 0.46 Hz to 4.04 ± 0.28 Hz, *p* = 1.09E-5) and 76.8% (from 9.88 ± 0.57 Hz to 7.58 ± 0.47 Hz, *p* = 1.94E-3) of baseline values. The mEPSC peak amplitude was also reduced to 87.4% (from 21.4 ± 0.5 pA to 18.7 ± 0.4 pA, *p* = 5.74E-5) and 81.5% (from 24.3 ± 0.6 pA to 19.8 ± 0.4 pA, *p* = 8.18E-10) of their baseline levels, respectively (Fig. 6*D−G*), consistent with what was observed following ACh or nicotine application (Figs. 4, 5).

### Blockade of Ca^2+^ internal stores occludes the effect of ACh on mEPSC frequency

**Figure 7:**
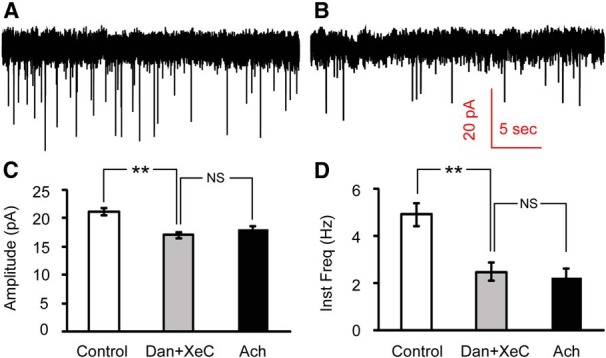
Inhibiting calcium release from internal stores occludes the effect of ACh on mEPSCs onto Hcrt+ neurons. ***A***, ***B***, mEPSCs recorded at baseline (***A***) and after 30 min incubation with the ryanodine receptor antagonist dantrolene (50 μM) and the IP3 receptor antagonist (−)-*Xestospongin C* (5 μM; ***B***). ***C***, ***D***, Mean values of amplitude (***C***) and instantaneous frequency (***D***) of mEPSCs at baseline, after 30 min incubation with dantrolene (Dan) and XeC and upon subsequent ACh puff in the continued presence of dantrolene and XeC*. n* = 3 cells. Both amplitude and instantaneous frequency were significantly decreased by dantrolene+XeC, but there was no significant difference between mEPSC recordings in the presence of dantrolene and XeC and upon subsequent ACh puffs in the continued presence of these inhibitors. **p < 0.01; NS, not significant (*p* > 0.05).

Synaptic vesicle release is a calcium-dependent process (Neher and Sakaba, 2008; Südhof, 2012). Spontaneous vesicle release, unlike action potential-evoked events, is independent of extracellular calcium and voltage-gated calcium channel opening, but is instead driven largely by calcium release from internal stores (Llano et al., 2000; Emptage et al., 2001; Han et al., 2001). To determine whether nAChR stimulation affects calcium release from internal stores, we applied dantrolene (50 μM) and (−)-Xestospongin C (XeC) (5 μM) to block ryanodine and IP_3_ receptors in the endoplasmic reticulum membrane (Dickinson et al., 2008). Following 30 min incubation with dantrolene and XeC, the mEPSCs instantaneous frequency was reduced to 50.7% of its baseline value (4.90 ± 0.48 Hz to 2.48 ± 0.38 Hz, *p* = 7.12E-4; Fig. 7), in agreement with previous reports that approximately half of spontaneous vesicle release is due to internal calcium stores (Emptage et al., 2001). The peak amplitude was reduced to 80.6% (21.1 ± 0.7 pA to 17.0 ± 0.5 pA, *p* = 5.18E-5). In the continued presence of dantrolene and XeC, we pressure applied ACh onto Hcrt+ neurons and found that both amplitude (from 17.0 ± 0.5 pA to 18.0 ± 0.5 pA, *p* = 0.664) and frequency of mEPSCs (from 2.48 ± 0.38 Hz to 2.25 ± 0.37 Hz, *p* = 0.151) remained unchanged (Fig. 7*C*,*D*). Thus, blockade of calcium release from internal store occluded the effect of subsequent nAChR stimulation.

## Discussion

Hypocretin/orexin neurons are involved in multiple behaviors, including those related to arousal and addiction (Mahler et al., 2012; de Lecea and Huerta, 2014). Understanding how the electrical activity of Hcrt+ neurons is modulated by endogenous neurotransmitters and exogenous drugs such as nicotine is critical for understanding the role of these cells in complex behaviors, including nicotine dependence. As a candidate modulator, nAChRs have been shown to be expressed in hypothalamus. Stimulation of these receptors can activate Hcrt+ neurons (Pasumarthi et al., 2006; Pasumarthi and Fadel, 2008; Morgan et al., 2013); however, the underlying mechanisms by which nAChRs modulate the activity of Hcrt+ neurons are still unclear.

In the current study, we used pressure application to mimic phasic ACh neurotransmission (Alexander et al., 2009) and investigated the effects of ACh and nicotine acting through presynatpic and postsynaptic nAChRs on glutamatergic transmission and postsynaptic electrical activity in Hcrt+ neurons. We found that nAChRs are expressed postsynaptically in around one-third of Hcrt+ neurons, as well as presynaptically on glutamatergic axon terminals to Hcrt+ neurons. Stimulation of presynaptic or postsynaptic nAChRs produced opposing effects on electrical activity in Hcrt+ neurons, however. Stimulation of postsynaptic nAChRs evoked an inward current in a fraction (approximately one-third) of Hcrt+ neurons (Fig. 3). This current, which was as small as 10 pA, ranging to more than 1000 pA, depolarized the membrane potential and increased spontaneous firing of Hcrt+ neurons (Fig. 1). In contrast, stimulation of presynaptic nAChRs reliably decreased the frequency of mEPSCs in Hcrt+ neurons (Fig. 4), indicating the efficacy of glutamatergic transmission was decreased. At a concentration consistent with cigarette smoking, nicotine suppressed mEPSCs in a majority (∼70%) of Hcrt+ neurons (Fig. 5). The nonselective nAChR antagonist mecamylamine depressed mEPSC frequency to a similar extent (Fig. 6), suggesting that the suppressing effect of ACh on mEPSCs was likely mediated by desensitization of presynaptic nAChRs, and that an endogenous, tonic activation of presynaptic nAChRs might be required for maintaining normal, functional mEPSC frequency. It should be noted that inhibitory inputs impinging on hypocretin neurons are also likely to express nAChRs, and therefore could also be regulated by ACh; however, inhibitory inputs were excluded using picrotoxin in this study.

To explore the mechanism underlying the ACh-mediated decrease in mEPSC frequency, we determined the role of calcium release from internal stores (Llano et al., 2000; Cheng and Lederer, 2008), since intracellular calcium stores are thought to be required for spontaneous vesicle release (Emptage et al., 2001). In the presence of the ryanodine receptor blocker dantrolene and the IP3 receptor blocker XeC (Dickinson et al., 2008), mEPSC frequencies in Hcrt+ neurons were significantly decreased. The application of ACh did not further decrease mEPSC frequencies (Fig. 7), indicating that phasic nAChR stimulation (i.e., endogenous ACh release from cholinergic synapses) could desensitize tonically active nAChRs and thereby decrease calcium release from internal stores, preventing Ca^2+^-dependent spontaneous vesicle release. Previous studies have shown that nicotine can transiently facilitate neurotransmitter release, and one study in mouse vas deferens indicated that this is mediated by calcium-induced calcium release from a ryanodine-sensitive calcium store in nerve terminals (Brain et al., 2001). Here, we propose that in the mouse central nervous system, at least in glutamatergic synapses impinging onto Hcrt+ neurons, this mechanism is also present. Tonic activation of presynaptic nAChRs contributes to spontaneous vesicle release via transient opening of ryanodine receptors and/or IP3 receptors in local internal calcium stores. Levels of nicotine delivered through cigarette smoking are sufficient to interfere with this function, and this mechanism could therefore contribute to behaviors related to nicotine dependence.

**Figure 8:**
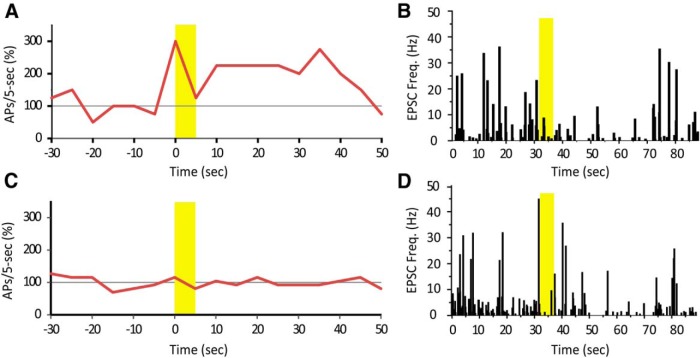
ACh increased action potential (AP) firing or had no effect. In the same cells, however, ACh decreased mEPSC occurrence despite of its effect on action potential firing. ***A***, A Hcrt+ cell increased action potential firing rate in response to ACh, in presence of atropine. ***B***, In the same cell, a puff of ACh decreased mEPSC frequency. Yellow bars indicate the time duration of ACh application. ***C***, Another Hcrt+ cell did not change action potential firing rate upon ACh puff. ***D***, In the same cell, a puff of ACh decreased mEPSC frequency.

The opening of nAChRs channels leads to the influx of cations, and generally excites the postsynaptic neurons (Léna and Changeux, 1997; Zhou et al., 2001; Mansvelder et al., 2002; Sharma et al., 2008; Huang et al., 2011). In the current study, this direct depolarizing effect of nAChR activation was also demonstrated for mouse Hcrt+ neurons, likely leading to hypocretin release in downstream neuronal circuits. The pharmacological experiments shown here, along with previous studies (Pasumarthi et al., 2006; Pasumarthi and Fadel, 2008; Morgan et al., 2013), suggest that α4β2* nAChRs are most critical for postsynaptic responses to ACh and nicotine in Hcrt+ neurons; postsynaptic α7 nAChRs also contribute to Hcrt+ nicotinic responses, but are less prominent. Blockade of α4β2* receptors by DHβE not only decreases mEPSCs by disrupting presynaptic nicotinic signaling, but also prevents the activation of postsynaptic nicotinic receptors, and therefore affects spontaneous firing of Hcrt+ neurons. In contrast, the role of presynaptic nAChRs has not been reported previously. In our experimental paradigm, either pressure application of ACh (1 mM) onto the soma and the proximal processes or bath application of nicotine (1 μM) suppressed the spontaneous mEPSC frequency in Hcrt+ neurons, which is consistent with effects reported in the arcuate nucleus of the hypothalamus (Huang et al., 2011). We also observed a consistent, though modest, reduction in mEPSC size that always accompanied the reduction in mEPSC frequency (Figs. 4 − 7). This could be due to effects of nicotinic signaling on activity of postsynaptic ionotropic glutamate receptors, in particular AMPA receptors. It is also possible that large presynaptic vesicles (Wojcik et al., 2004) or proximal synapses (which produce mEPSCs with larger amplitudes than distal ones; Bekkers and Stevens, 1996) are more significantly recruited by nicotinic signaling. Interestingly, the nicotinic antagonist, MEC, had a similar effect as ACh and nicotine on mEPSC frequency and amplitude (Figs. 4 − 6). Partial blockade of α4β2 or α7 nAChRs by more selective antagonists also reduced mEPSC frequency, indicating that both heteromeric and homomeric nAChRs in brain are present in presynaptic glutamatergic terminals impinging on Hcrt+ neurons. These results suggest that both ACh and nicotine likely desensitize nAChRs and interrupt glutamatergic transmission onto Hcrt+ neurons, further suggesting that tonic activation of presynaptic nAChRs might be necessary for the normal function of neurotransmission in glutamatergic synapses impinging onto these neurons (Brain et al., 2001). Phasic cholinergic release may temporarily interrupt or reduce this tonic glutamatergic transmission. It is also possible that stimulation of postsynaptic nAChRs depolarizes membrane potential and releases calcium from internal stores, facilitating release of retrograde signaling molecules, such as endocannabinoids (Huang et al., 2007) or dynorphin (Li and van den Pol, 2006), that result in negative feedback. These paradoxical presynaptic and postsynaptic effects of ACh may improve the signal-to-noise ratio of selective Hcrt+ firing during phasic release, as might occur during exposure to behaviorally relevant stimuli (Dalley et al., 2001; Parikh et al., 2007), or in response to nicotine during smoking or behaviors relevant to drug reinforcement (Hollander et al., 2008). In particular, phasic ACh release would be expected to stimulate the firing of the approximately one-third of Hcrt+ neurons expressing nAChRs, while also reducing activity in the remaining two-thirds of Hcrt+ neurons by blocking synaptic excitatory events supported by tonic activation of presynaptic nAChRs (Fig. 8).

In conclusion, these results suggest that ACh modulates the Hcrt system through a multifaceted, nAChR-mediated mechanism, which is complementary to the effects of muscarinic modulation of these neurons (Sakurai et al., 2005). More importantly, nAChR-mediated effects of ACh may enhance the output of a selective group of Hcrt+ neurons expressing nAChRs, and distinguish this subset from the overall hypocretin network.

## Synthesis

The decision was a result of the Reviewing Editor Liset Menendez de la Prida and the peer reviewers coming together and discussing their recommendations until a consensus was reached. A fact-based synthesis statement explaining their decision and outlining what is needed to prepare a revision is listed below. The following reviewers agreed to reveal their identity: Washington Buno

Both reviewers appreciate the ms touches an interesting topic in the field, by directly addressing the mechanisms of action of nicotinic receptors in hypocretine hypothalamic neurons, a critical cell type involved in signaling rewarding and addiction. However, for one reviewer (R2) the scope is a bit narrow and somehow anticipated by previous publications. So, it may be advisable that you revise your ms with this in mind to try bringing novelty and impact more in focus. Please, keep in mind eNeuro looks for research papers that contribute to field of neuroscience by significantly expanding knowledge. In addition, you will need to perform additional control experiments and analysis to clarify on some specific points raised by reviewers, which I detail below. These experiments seem to require less than 2 months' work, which is a critical threshold for decision-making in eNeuro. We hope our comments and suggestions will help you to improve this potentially interesting manuscript.

Major concerns:

1- A fundamental question that is posed by R2 is regarding the identity of cells exhibiting opposing trends of firing responses to ACh which is a fundamental conclusion of the ms. You claim ACh acts to increase firing rate in 1/3 of Hcrt+ cells by postsynaptic mechanisms, while decrease firing rate of the remaining 2/3 by decreasing presynaptic activity, but they never tested both groups at once. This can be directly tested by obtaining cell-attached recordings to first validate firing modulation in response to ACh puffs and then examine changes of mEPSC frequency and amplitude.
2- A connected issue points to mechanical effect of the puffs, which confound data interpretation and numbers. In particular, R2 commented a potential disagreement of numbers as reported in first experiments in Fig.1D that suggest only 9/20 cells exhibited firing decrease (presumably presynaptically modulated according to authors' conclusion). This number is comparable to data from ACSF puffs (Fig.1C) and does not match with the 2/3 authors claim. You need to further discuss this. Are your experimental conditions suitable to detect firing decreases?
3- A second major issue was commented by R1 and is regarding a generalization of the effect of ACh (and nicotine) in more realistic in vivo like conditions. Actually, I feel points i, ii and vii by R1 touch this point similarly. In point (i) R1 asks about the effect of ACh puffs (and/or nicotine) when atropine is not present (i.e., whether the effect of muscarinic receptors matters). Point (ii) asks for circuit effect of ACh/nicotine acting through GABAergic interneurons, while point (vii) asks for controlling cell responses in normal ACSF without blockers.
  To address this point, you need to clarify whether experiments in Fig.1D were performed in the presence of blockers of synaptic transmission (apart from atropine), as also asked by R2. If so you need to perform a set of experiments in standard ACSF without any added blocker to evaluate the overall effect of ACh/nicotine in in vivo like conditions. This point is intimately connected with point 2 above.

Other comments include:

4- Point (iii) by R1 asks about confounding effect of endocannabinoid release activated by direct ACh depolarization of terminals, which may affect interpretation of mEPSC data (Huang et al. JN 2007 describes CB1-mediated inhibition of Hcrt cells by presynaptic attenuation of glut release). Point (iv) consider an indirect effect caused by high concentration of patch pipette ATP required for some experiments, which could transform into adenosine. Adenosine would then confound interpretation of mEPSP by affecting presynaptic release (Liu and Gao JNeurophysiol 2007 shows adenosine decrease firing reate of Hcrt cells by acting presynaptically). You need to carefully discuss/discard these possible confounding factors in your data.
5- The remaining concerns can be addressed by further analysis, clarifications and discussion, not requiring experiments. In particular, data presented in Fig.1E should be analyzed statistically to address point by R2 regarding reduction of firing rate by ACh puffs in the presence of DhbetaE. Authors also need to clarify whether application of DhbetaE or MLA change firing rate of the Hcrt+ cells prior to the ACh puff.

Some other minor points and clarifications include:

6- Authors should also discuss their data on reduction of mEPSC amplitude and their interpretation as a postsynaptic effect, as suggested by R2, and also mentioned by R1 (point vi). What is causing this decrease in amplitude? Do the authors believe there is a postsynaptic change in glutamate receptors?
7- Point (vi) by R1 regarding absence of any desensitization effect on ACh-induced firing should be further clarified by authors.
8-As suggested by R2, they should mention pipette resistance and recording conditions (series resistance) in Methods
9-Colors used in Fig.1 should be clarified
10-Need to specific how long instantaneous freq were analyzed (last point by R2)

### R1's comments:

This manuscript reports that in the hypothalamus of mice, neurons expressing the neuropeptide hypocretin are excited by acetylcholine and nicotine puffs applied close to the soma in the presence of atropine. These puff that act through nicotinic receptors and could induce an inward current. By contrast, acetylcholine and nicotine puffs also reduced the frequency and amplitude of miniature EPSCs, a presynaptic action that was suppressed by blocking Ca2+ release from internal stores. The manuscript concludes that these opposing pre- and postsynaptic effects could functions to increase the signal-to-noise ratio in responses evoked by phasic release of acetylcholine.

The work provides interesting and convincing evidence that elucidate the action of nicotinic receptors on the pre and postsynaptic regulation of neurons expressing the neuropeptide hypocretin and discusses how this nicotinic regulation could contribute to behavior and cigarette smoking or other addictive behaviors.

However several aspects pointed below deserve attention:

(i) Although the authors mention that muscarinic receptors are also functional in these orexin neurons (lines 340-341), however they do not show not or discuss how the combined effects of acetylcholine and nicotine puffs would regulate firing and miniature EPSC, a central issue to understand the functional relevance of the cholinergic regulation.
(ii) These cells also receive inhibitory inputs; e.g., Schöne et al., 2011; Henny et al., 2006; Baldo et al., 2004 that could also be regulated by acetylcholine, a point that should be investigated or at least discussed (miniature EPSC were recorded under picrotoxin).
(iii) The ACh mediated depolarization and Ca2+ release from internal stores could induce endocannabinoid release and contribute to the presynaptic regulation.
(iv) Although the reason for a high concentration of ATP is mentioned (lines 340-341), this ATP could be released and converted into adenosine presynaptically inhibit remitter release.
(v) It should be clarified when and why ionotropic glutamate receptors were blocked.
(vi) The authors should indicate why in Fig. 1B2, D and E, ACh puffs induce prolonged increases in firing without apparent desensitization and why miniature EPSC amplitude was modified.
(vii) It would be highly revealing if the authors perform experiments in relevant physiological conditions, under current-clamp, without blocking GABA or glutamate receptors, while stimulating cholinergic inputs.

### R2's comments:

The manuscript is clearly written and easy to understand. The topics covered are interesting; however, the overall impact is somewhat limited due to the narrow scope of the manuscript. The authors do a nice job of showing data to support their claim that nicotinic receptors have an important role in regulating the activity of hypocretin neurons, although some of this work has already been published by the authors and/or others. In addition, there are some points that require additional clarification:

In the methods section there is no mention of the pipette resistance of the recording pipette or the acceptable range of series resistance used during experiments.

In the discussion the authors make a statement that nicotine may increase the firing rate of 1/3 of Hcrt+ cells while reducing activity in the other 2/3 by decreasing excitatory input. However, this conclusion is not supported by their data- it was never shown that the cells that show an increase in firing are distinct from the cells that have a reduction in glutamatergic input.

Were the experiments examining firing rate performed in the presence of blockers of synaptic transmission? If not, then the authors' argument in the discussion section that the decrease in mEPSC frequency elicited by the ACh puff would decrease activity in 2/3 of Hcrt+ neurons is refuted, given that only 9 of 20 cell showed a decrease in firing rate, and this percentage is similar to what is observed with only an ACSF puff.

The data presented in figure 1E seem to show that in the presence of DhβE there was a dramatic and sustained reduction in firing rate induced by the ACh puff- more so than observed with just the ACSF puff. The authors make no mention of this decline in firing rate and whether or not it was statistically significant. Could the authors comment on this effect?

Does the application of DhβE or MLA change firing rate of the Hcrt+ cells prior to the ACh puff?

A reduction in mEPSC amplitude usually indicates a postsynaptic effect. However, the authors do not discuss what they believe is causing this decrease in amplitude. Do the authors believe there is a postsynaptic change in glutamate receptors?

Define for how long "instantaneous frequencies" were analyzed.

## Author Response

We thank the editor and the reviewers for these timely, comprehensive and fair reviews. We have made every effort to address the critiques thoroughly, and we believe that the revised version is substantially improved. In the revised manuscript, new text is marked in blue font to help the reviewers identify changes to the manuscript. Below, we address each critique individually.

### Editor’s Synthesis:

The species studied is not mentioned in the abstract. Please make sure to update both the abstract in the article file and on the submission form.

The species studied (mouse) has been added to the Abstract.

In addition, to address the concern of Reviewer 2 that the scope of the original version was somewhat narrow, we have added text to the Introduction (lines 60-67) to put the current study into the context of nicotine addiction. Further, we hope the addition of new experiments will also respond to this concern. New data have been incorporated into the previous figures and 3 new figures have been added to the revised manuscript to incorporate the data requested by the reviewers.

1. A fundamental question that is posed by R2 is regarding the identity of cells exhibiting opposing trends of firing responses to ACh, which is a fundamental conclusion of the ms. You claim ACh acts to increase firing rate in 1/3 of Hcrt+ cells by postsynaptic mechanisms, while decrease firing rate of the remaining 2/3 by decreasing presynaptic activity, but you never tested both groups at once. This can be directly tested by obtaining cell-attached recordings to first validate firing modulation in response to ACh puffs and then examine changes of mEPSC frequency and amplitude.
  As suggested, we conducted a new set of experiments to address this question. In the same Hcrt+ cell, we first puffed ACh during current clamp to determine the effect on modulation of firing. Next, we bath applied TTX+PTX and puffed ACh to examine changes in mEPSC frequency (new Fig. 8). Again we observed two types of firing modulation, increase or decrease/no change (new Fig. 8A&C). However, in the same cell, no matter in which direction firing was modulated, a puff of ACh reliably reduced mEPSC frequency (new Fig. 8B&D). (Note that mechanical interference may also decrease firing rate. Therefore we would classify firing decrease and no change as a similar response.) Activation of nAChRs in the postsynaptic cell induces an inward current and directly depolarizes the cell, which is a much stronger drive and dominates over presynaptic modulation.
2. A connected issue points to mechanical effect of the puffs, which confound data interpretation and numbers. In particular, R2 commented a potential disagreement of numbers as reported in first experiments in Fig. 1D that suggest only 9/20 cells exhibited firing decrease (presumably presynaptically modulated according to authors' conclusion). This number is comparable to data from ACSF puffs (Fig.1C) and does not match with the 2/3 authors claim. You need to further discuss/test this. Are your experimental conditions suitable to detect firing decreases?
  We agree that the experimental design is not suitable for detecting decreases in firing due to presynaptic mechanisms, which we also now show is dominated by postsynaptic mechanisms in the slice condition. Li et al. have demonstrated that only by blocking all mEPSCs through bath application of CNQX+AP-5 can one mildly change the firing rate of a hypocretin neuron (∼2 Hz to ∼1.6 Hz) (Neuron. 2002 36(6):1169-81), which is not distinguishable using our criteria (50% < change < 200%). In our case, ACh was applied only to the soma and the proximal processes, and its efficacy was far less strong compared to bath application of CNQX+AP-5. Changes caused by mechanical interference could easily obscure the presynaptic effects. We have added a discussion of these points to the manuscript, lines 157-161.
3. A second major issue was commented by R1 and is regarding a generalization of the effect of ACh (and nicotine) in more realistic in vivo like conditions. Actually, I feel points i, ii and vii by R1 touch this point similarly. In point (i) R1 asks about the effect of ACh puffs (and/or nicotine) when atropine is not present (i.e. whether the effect of muscarinic receptors matters). Point (ii) asks for circuit effect of ACh/nicotine acting through GABAergic interneurons, while point (vii) asks for controlling cell responses in normal ACSF without blockers. To address this point, you need to clarify whether experiments in Fig.1D were performed in the presence of blockers of synaptic transmission (apart from atropine), as also asked by R2. If so you need to perform a set of experiments in standard ACSF without any added blocker to evaluate the overall effect of ACh/nicotine in in vivo like conditions. This point is intimately connected with point 2 above.
  The experiments shown in Fig. 1D were performed in presence of atropine, picrotoxin, CNQX and AP-5. To address this concern, we conducted a new set of experiments in the absence of atropine and synaptic blockers to allow the activation of both nicotinic and muscarinic receptors. Our results show that Ach still reduced mEPSC frequency in the absence of atropine, similarly to what was recorded in the presence of atropine (data not shown). On the other hand, ACh increased spontaneous firing rate in 57% of cells when atropine was removed, compared to 35%, in presence of atropine (new Fig. 1G). Chi square test indicate the difference is significant (p=0.0040). These results suggest that muscarinic receptors recruit activity of an additional proportion of Hcrt cells in response to cholinergic stimulation, which is consistent with the published results on the effects M type ACh receptors on hypocretin neurons.
  The related questions asked by Reviewers 1 and 2 are addressed below. New data have been added to the Results section, lines 183-190.
4. Point (iii) by R1 asks about confounding effect of endocannabinoid release activated by direct ACh depolarization of terminals, which may affect interpretation of mEPSC data (Huang et al. JN 2007 describes CB1-mediated inhibition of Hcrt cells by presynaptic attenuation of glut release). Point (iv) consider an indirect effect caused by high concentration of patch pipette ATP required for some experiments, which could transform into adenosine. Adenosine would then confound interpretation of mEPSP by affecting presynaptic release (Liu and Gao JNeurophysiol 2007 shows adenosine decrease firing rate of Hcrt cells by acting presynaptically). You need to carefully discuss/discard these possible confounding factors in your data.
  We agree that multiple mechanisms may contribute to the effect we observed in this study. We now discuss the possibility of the involvement of endocannabinoid and dynorphin signaling, although we excluded the possibility that adenosine contributes to the current findings because it is present at much lower levels. See lines 371-374. Current data supported the inhibition of hypocretin neurons by bath application of adenosine or endogenous adenosine resulting from the activity of a network of neurons. However, in either case, the level of adenosine is likely much higher than what may be produced from a single cell. The including of a relatively high level of ATP in the pipette solution is due to the reason that hypocretin neurons may require a high ATP level *in situ*, which is usually impaired by the conventional whole cell recording (see Liu et al., 2011).
5. The remaining concerns can be addressed by further analysis, clarifications and discussion, not requiring experiments. In particular, data presented in Fig.1E should be analyzed statistically to address point by R2 regarding reduction of firing rate by ACh puffs in the presence of DhbetaE. You also need to clarify whether application of DhbetaE or MLA change firing rate of the Hcrt+ cells prior to the ACh puff.
  We performed additional statistical analysis for Fig. 1E, now included in the Results section. As suggested, we tested the effects of DhβE and MLA alone on the change in firing rate. We found that the firing rate was significantly reduced by DhβE alone (20-30s after puff vs. 20 to10s before puff, new Fig. 2A, p=0.00027), but not by MLA. We believe that it is reasonable to use these agents to identify the contribution of particular nAChR subtypes because most cells recovered to a new stable firing state that lasted for several minutes with the attachment of the recording pipette (Fig. 2B) following the addition of either reagent. Furthermore, in the experiments shown in Fig. 1, DhβE or MLA was added in the bath many minutes before patching into the cells. Not being attached to a patch pipette makes a cell much healthier.
6. You should also discuss data on reduction of mEPSC amplitude and interpretation as a postsynaptic effect, as suggested by R2, and also mentioned by R1 (point vi). What is causing this decrease in amplitude? Do you believe there is a postsynaptic change in glutamate receptors?
  We agree with the reviewers that some postsynaptic and/or other mechanism (s) might have caused the reduction of mEPSC amplitude. We have added more on this point to the Discussion section (lines 357-361).
7. Point (vi) by R1 regarding absence of any desensitization effect on ACh-induced firing should be further clarified.
  We consider that the activation of recurrent circuitry by nicotinic signaling might have caused a prolonged increase in firing rate by ACh. We have added a sentence on this point to the manuscript, lines 178-182.
8. As suggested by R2, you should mention pipette resistance and recording conditions (series resistance) in Methods
  We have added these important methodological details to the Methods on lines 109-113.
9. Colors used in Fig. 1 should be clarified
  This has been corrected.
10. Need to specific how long instantaneous freq were analyzed (last point by R2)
  This information has been added on lines 133-136.

### Reviewer #1

(i) Although the authors mention that muscarinic receptors are also functional in these orexin neurons, they do not show not or discuss how the combined effects of acetylcholine and nicotine puffs would regulate firing and miniature EPSC, a central issue to understand the functional relevance of the cholinergic regulation.
  We agree with the reviewer that muscarinic receptors likely impact ACh-mediated changes in firing and/or mEPSCs in hypocretin neurons. We therefore conducted both experiments in the absence of atropine to allow the activation of both nicotinic and muscarinic receptors. Our results show that ACh still reduced mEPSC frequency in the absence of atropine, without a statistical difference compared to recording in the presence of atropine (data not shown). On the other hand, ACh increased the spontaneous firing rate in 57% of cells when atropine was removed, compared to 35%, in presence of atropine (new Fig. 1G). A Chi square test indicates that the difference is significant (p=0.0040). This experiment suggests that muscarinic receptors help engage a bigger proportion of Hcrt cells to increase their firing rate in response to cholinergic stimulation. See lines 183-190 in the Results.
  Here we also want to clarify that our focus in this paper is on whether nicotine in tobacco might alter firing of Hcrt neurons, and therefore we put most of the emphasis on nicotinic acetylcholine receptors. We used acetylcholine combined with atropine as a tool to stimulate nAChRs because it allows better ligand-receptor interaction (compared to puffing lipophilic nicotine onto brain slice), and allows faster receptor recovery from desensitization.
(ii) These cells also receive inhibitory inputs; e.g., Schöne et al., 2011; Henny et al., 2006; Baldo et al., 2004 that could also be regulated by acetylcholine, a point that should be investigated or at least discussed (mEPSC were recorded under picrotoxin).
  We agree that those inhibitory inputs impinging on hypocretin neurons are likely to express nicotinic (and/or muscarinic) receptors and therefore be regulated by ACh. We excluded inhibitory inputs by using picrotoxin in this study because we were originally interested in feed forward processes that might increase firing of Hcrt neurons. While this is an important point, we have not focused further on the GABA inputs and we now mention that this aspect of presynaptic function could also be affected in the Discussion, lines 315-318.
(iii) The ACh mediated depolarization and Ca2+ release from internal stores could induce endocannabinoid release and contribute to the presynaptic regulation.
  We agree it is possible (perhaps likely) that neuromodulators such as endocannabinoids or dynorphin might mediate the nicotinic effects on mEPSCs presynaptically. We have added this point to the Discussion, lines 371-374.
(iv) Although the reason for a high concentration of ATP is mentioned, this ATP could be released and converted into adenosine presynaptically to inhibit transmitter release.
  In this experiment, ATP is only added in the intracellular solution in the patch pipette at the cell body. A very limited amount of ATP is therefore available to reach surrounding axon terminals either through release or as a result of leakage. Therefore, we do not believe that adenosine can reach the effective concentration (100 µM, Liu & Gao, 2007) that might confound the interpretation of the nicotinic effect on mEPSC frequency.
(v) It should be clarified when and why ionotropic glutamate receptors were blocked.
  This information has been added in lines 198-199.
(vi) The authors should indicate why in Fig. 1B2, D and E, ACh puffs induce prolonged increases in firing without apparent desensitization and why miniature EPSC amplitude was modified.
  This is an interesting question and we have added discussion of some possibilities to the revised manuscript. In this data set, subsets of hypocretin neurons showed very different patterns of firing as a result of nicotinic stimulation (Fig. 1D, F&G): some cells increased their firing rate during the ACh puff and returned normal right after puff cessation (7 out of 17); the other cells responded with a prolonged period of firing increase (10 out of 17). We consider that in the former cells, the response pattern is clearly consistent with desensitization of nAChRs. In the latter cells, it is likely that the boosted firing of Hcrt neurons triggered action potentials in a recurrent circuit, resulting in a prolonged period of firing.
  We have added a discussion of this point to the revised manuscript (lines 178-182).
(vii) It would be highly revealing if the authors perform experiments in relevant physiological conditions, under current-clamp, without blocking GABA or glutamate receptors, while stimulating cholinergic inputs.
  We agree that an experiment based on the stimulation of endogenous cholinergic inputs (e.g., optogentics) would be very useful in the understanding of the modulation of the hypocretin system by the cholinergic system, which warrants further investigation along this direction. However, the scope of current study aims to address the effects of nicotine receptors on hypocretin neurons in a context of the nicotine use. Therefore, we partially addressed this question by adding data to the manuscript at the suggestion of the editor and reviewers in the absence of atropine or other blockers to at least engage all the ACh receptor populations. The results of this new experiment are informative about the proportion of Hcrt neurons likely to be engaged as a result of ACh release, and have been added to the manuscript.

### Reviewer #2

(i) In the methods section there is no mention of the pipette resistance of the recording pipette or the acceptable range of series resistance used during experiments.
  This information has been added to lines 109-113.
(ii) In the discussion the authors make a statement that nicotine may increase the firing rate of 1/3 of Hcrt+ cells while reducing activity in the other 2/3 by decreasing excitatory input. However, this conclusion is not supported by their data- it was never shown that the cells that show an increase in firing are distinct from the cells that have a reduction in glutamatergic input. Were the experiments examining firing rate performed in the presence of blockers of synaptic transmission? If not, then the authors' argument in the discussion section that the decrease in mEPSC frequency elicited by the ACh puff would decrease activity in 2/3 of Hcrt+ neurons is refuted, given that only 9 of 20 cell showed a decrease in firing rate, and this percentage is similar to what is observed with only an ACSF puff.
  First, we want to clarify that our statement that “nicotine may increase the firing rate of 1/3 of Hcrt+ cells while reducing activity in the other 2/3 by decreasing excitatory input” is based on our results that around 1/3 of Hcrt+ cells responded to ACh with an increase in firing rate or with an inward current, meanwhile all recorded Hcrt+ cells showed a decrease in mEPSC frequency. We did not test the idea with the experiments shown in Fig. 1. Actually, the experiments were neither designed, nor suitable for the purpose. This is because mEPSC is generally a weak factor contributing to action potential firing. Li et al. has demonstrated that only by blocking all mEPSCs (CNQX+AP-5) can one mildly change the firing rate of a hypocretin neuron (∼2 Hz to ∼1.6 Hz) (Neuron. 2002 36(6):1169-81), which is not distinguishable using our criteria (50% < change < 200%). In this set of experiments, synaptic blockers were present, including picrotoxin, CNQX, AP-5 and atropine.
  In the revised manuscript we present a new set of experiment in which we first puffed ACh onto Hcrt+ cells in the presence of atropine during current clamp to establish the ability of ACh to modulate firing rate. We next we bath applied TTX+PTX and puffed ACh onto the same cell to examine changes in mEPSC frequency (new Fig. 8). Again, we observed two types of firing modulation: increase and decrease/no change; however, in the same cell, no matter how firing was modulated, a puff of ACh reliably reduced mEPSC frequency. (Note that mechanical interference may also decrease firing rate, therefore we would classify firing decrease and no change as a similar response.). We think that an activation of nAChRs in the postsynaptic cell induces an inward current and directly depolarizes the cell, which is a much stronger factor and dominates over the changes in presynaptic input.
(iii) The data presented in figure 1E seem to show that in the presence of DhβE there was a dramatic and sustained reduction in firing rate induced by the ACh puff- more so than observed with just the ACSF puff. The authors make no mention of this decline in firing rate and whether or not it was statistically significant. Could the authors comment on this effect?
  It is true that in the presence of DhβE, an ACh puff was more likely to decrease the firing rate. As suggested, we conducted a t-test and the difference is highly significant (10-20s after puff, p = 6.8E-6). This has been added to the manuscript in the Results section (line 174-175). We think this suggests that α4β2* receptors are important for ACh-mediated modulation of activity in Hcrt+ neurons (Fig. 1E&F; 5E&G). Blockade of α4β2* receptors by DhβE not only decreases mEPSCs by disrupting presynaptic nicotinic signaling, but also prevents the activation of postsynaptic nicotinic receptors, and therefore affects spontaneous firing of Hcrt+ neurons. This information has been added to the manuscript, lines 348-350.
(iv) Does the application of DhβE or MLA change firing rate of the Hcrt+ cells prior to the ACh puff?
  We conducted a new set of experiments to address this point as suggested (lines 168-173). MLA did not change the firing rate of Hcrt neurons at a concentration of 10 nM (new Fig. 2. 20-30s after puff vs. 10 - 20s before puff, p=0.58); DhβE significantly decreased the firing rate by 62.9% (p=0.00027) at 10 µM. However, after addition of either reagent, most cells recovered to a new stable firing state that lasted for several minutes with attachment of the recording pipette (Fig. 2B). Furthermore, in the experiments shown in Fig. 1, DhβE or MLA was added to the bath long before patching into the cells. Not being attached to patching pipette makes a cell much healthier.
(v) A reduction in mEPSC amplitude usually indicates a postsynaptic effect. However, the authors do not discuss what they believe is causing this decrease in amplitude. Do the authors believe there is a postsynaptic change in glutamate receptors?
  We agree with the reviewer that mEPSC amplitude might be affected by postsynaptic factors. We have added an explanation of this point to the Discussion, lines 357-361.
(vi) Define for how long "instantaneous frequencies" were analyzed.
  For baseline and washout, we took 150-200 events from each cell, during a time period ranging 60-120 sec. For ACh we took events from within 90 sec after application of ACh. We have added this information to the Materials and Methods, lines 133-136.
